# Acoustic radiation force impulse (ARFI) elastography compared with biopsy for evaluating hepatic fibrosis after liver transplantation: a cross-sectional diagnostic study: Elastografia ARFI (*acoustic radiation force impulse* ) comparada com biópsia na avaliação de fibrose hepática após transplante: estudo transversal diagnóstico

**DOI:** 10.1590/1516-3180.2016.0158170816

**Published:** 2016-06-03

**Authors:** Joel Schmillevitch, Maria Cristina Chammas, Vincenzo Pugliese, Edson Abdala, Adriana Cortez Rizzon, Venâncio Alves, Luiz Augusto Carneiro, Giovanni Cerri

**Affiliations:** I MD. Researcher, Instituto de Radiologia (InRad), Hospital das Clínicas (HC), Faculdade de Medicina da Universidade de São Paulo (FMUSP), São Paulo, SP, Brazil.; II MD, PhD. Director, Ultrasonography Service, Instituto de Radiologia (InRad), Hospital das Clínicas (HC), Faculdade de Medicina da Universidade de São Paulo (FMUSP), São Paulo (SP), Brazil.; III MD, PhD. Attending Physician, Liver and Digestive System Organ Transplantation Division, Hospital das Clínicas (HC), Faculdade de Medicina da Universidade de São Paulo (FMUSP), São Paulo (SP), Brazil.; IV MD, PhD. Professor, Department of Infectious and Parasitic Diseases, Hospital das Clínicas (HC), Faculdade de Medicina da Universidade de São Paulo (FMUSP), São Paulo (SP), Brazil.; V Nurse, Coordinator of the Liver and Digestive System Organ Transplantation Division, Hospital das Clínicas, Faculdade de Medicina da Universidade de São Paulo (FMUSP), São Paulo (SP), Brazil.; VI MD, PhD. Titular Professor, Department of Pathology, Faculdade de Medicina da Universidade de São Paulo (FMUSP), São Paulo (SP), Brazil.; VII MD, PhD. Titular Professor and Director, Liver and Digestive System Organ Transplantation Division, Hospital das Clínicas (HC), Faculdade de Medicina da Universidade de São Paulo (FMUSP), São Paulo (SP), Brazil.; VIII MD, PhD. Titular Professor, Department of Radiology, Universidade de São Paulo (FMUSP), São Paulo (SP), Brazil.

**Keywords:** Elasticity imaging techniques, Liver transplantation, Fibrosis, Liver cirrhosis, Ultrasonography, Técnicas de imagem por elasticidade, Transplante de fígado, Fibrose, Cirrose hepática, Ultrassonografia

## Abstract

**CONTEXT AND OBJECTIVE:**

Biopsies are used after liver transplantation to evaluate fibrosis. This study aimed to evaluate the elasticity of transplanted livers by means of a non-invasive examination, acoustic radiation force imaging (ARFI) elastography, correlating the results with the histological analysis.

**DESIGN AND SETTING:**

Cross-sectional study in a public university hospital.

**METHODS:**

All patients consecutively operated between 2002 and 2010 with an indication for biopsy were evaluated by means of elastography. The radiologist evaluating ARFI and the pathologist doing anatomopathological examinations were blinded to each other's evaluations.

**RESULTS:**

During the study period, 33 patients were included. The indication for transplantation was cirrhosis due to hepatitis C in 21 cases (63%). Liver biopsies showed absence of fibrosis (F0) in 10 patients, F1 in 11, F2 in 8 and F3 in 4. There were no cases of F4 (cirrhosis). The difference in ARFI values (degree of fibrosis) was 0.26 (95% confidence interval, CI: 0.07-0.52) between the groups F0-F1 and F2-F4 (P = 0.04). An area under the curve of 0.74 (CI: 0.55-0.94) and a cutoff of 1.29 m/s between the groups resulted in the best relationship between sensitivity and specificity. Sensitivity (0.66; CI: 0.50-0.83) was lower than specificity (0.85; CI: 0.72-0.97). There was no significant difference in ARFI between patients with hepatitis C and those with other diseases.

**CONCLUSIONS:**

The values obtained from elastography were not affected by inflammatory reaction or anatomical alterations. A cutoff point of 1.29 m/s separating patients with or without significant fibrosis was identified.

## RESUMO

**CONTEXTO E OBJETIVO:**

Biópsias são utilizadas para avaliar fibrose após transplante de fígado. O estudo objetivou avaliar a elasticidade hepática após transplante por meio de um exame não invasivo, a elastografia ARFI (*acoustic radiation force imaging* ), correlacionando-a com a análise histológica.

**TIPO DE ESTUDO E LOCAL:**

Estudo transversal em hospital público universitário.

**MÉTODOS:**

Todos os pacientes consecutivamente operados entre 2002 e 2010, com indicação para biópsia, foram avaliados por elastografia. O radiologista avaliando ARFI e o patologista fazendo exames anatomopatológicos estavam cegos para as avaliações um do outro.

**RESULTADOS:**

No período do estudo, 33 pacientes foram incluídos. A indicação para o transplante foi cirrose por hepatite C em 21 (63%). As biópsias mostraram ausência de fibrose (F0) em 10 pacientes, F1 em 11, F2 em 8, F3 em 4 e nenhum caso de F4 (cirrose). A diferença nos valores de ARFI (grau de fibrose) foi de 0,26 (intervalo de confiança, IC, de 95%: 0,07-0,52) entre os grupos F0-F1 e F2-F4 (P = 0,04). A área sob a curva de 0,74 (IC: 0,55-0,94) e o valor de corte de 1,29 m/s entre os grupos resultaram na melhor relação entre sensibilidade e especificidade, de 0,57. A sensibilidade (0,66; IC: 0,50-0,83) foi menor que a especificidade (0,85; IC: 0,72-0,97). Não houve diferença significativa em ARFI entre pacientes com hepatite C e aqueles com outras doenças.

**CONCLUSÕES:**

Os valores obtidos com a elastografia não foram afetados por reação inflamatória ou alterações anatômicas. Foi identificado ponto de corte de 1,29 m/s que separa pacientes com ou sem fibrose significativa.

## INTRODUCTION

Liver transplantation is an established form of therapy for treating acute and chronic end-stage hepatic diseases and for some hepatic tumors, with ten-year graft survival greater than 70% at most transplantation centers.^1^^,^^2^^,^^3^^,^^4^ Nonetheless, histological changes in transplanted livers can occur subclinically and progressively even in asymptomatic patients and without significant laboratory alterations.^3^^,^^4^^,^^5^^,^^6^

Liver biopsy is currently the gold standard for histological evaluation of the liver.^7^^,^^8^^,^^9^^,^^10^However, it is invasive and poses a risk of complications with morbidity around 0.3 to 0.6% and mortality of 0.05%, besides hospitalization of at least 6-18 hours.^3^^,^^4^ In order to diagnose these alterations, some transplantation centers recommend routine biopsies at fixed intervals (annually) after liver transplantation, thus called "protocol biopsies".^11^ Other centers do not follow this protocol, taking the view that liver biopsy is both risky and invasive.^3^^,^^4^

The main histological alterations observed in anatomopathological evaluations on biopsies on transplanted livers involve inflammation and fibrosis.^6^ However, the degree of fibrosis may be underestimated in situations of inadequate sampling, given that the volume of the sample evaluated in the biopsy is only 1/50,000 of the organ. There have been reports of discrepancies in both inter and intraobserver histological analyses, at estimated rates of 10 to 30% of the cases.^12^

New noninvasive diagnostic methods, such as serum tests, magnetic resonance imaging and elastography, which have shown significant correlation with the histological test,^13^ can be introduced with the objective of reducing the number of biopsies. This has already been happening in relation to patients with chronic hepatopathy. Several noninvasive methods have been proposed for staging hepatic fibrosis. These include indices based on biochemical tests and imaging examinations.^14^ Among the biochemical tests, the aspartate aminotransferase (AST) to platelet ratio index (APRI) stands out due to its simplicity and low cost. The APRI was proposed in 2003 by Wai et al. and promising results were reported.^15^^,^^16^

Transient hepatic elastography (Fibroscan, Echosens, France) was the first noninvasive diagnostic method for quantifying the degree of hepatic fibrosis, and the first scientific paper on this method was published in 2003.^17^ Its use on patients with liver disease is based on the relationship between stiffness and hepatic fibrosis. It measures the elasticity of a volume of hepatic tissue corresponding to a cylinder with a thickness of 1 cm and depth of 2 cm, with results expressed in kilopascals. Different studies have assessed its diagnostic accuracy. Using optimized cutoff points, Castéra et al.^7^ found a positive predictive value (PPV) of 95% and a negative predictive value (NPV) of 48% for the presence of significant fibrosis, and PPV of 77% and NPV of 95% for the presence of cirrhosis. The same group also reported concordance of 83% with liver biopsy regarding the diagnosis of significant fibrosis and 90% regarding the diagnosis of cirrhosis.^7^

Another method for quantifying the degree of hepatic fibrosis was presented more recently, in 2009: acoustic radiation force imaging (ARFI) (S2000, Siemens, Germany). In the ARFI elastography method, short-duration pulses are emitted in the region of interest (ROI), thus generating waves with velocities measured in meters per second.^18^^,^^19^ Fierbinteanu-Braticevici et al. measured the area under the ROC curve of ARFI elastography and found accuracy of 90.2% in predicting fibrosis.^20^ Some advantages of ARFI elastography merit special emphasis, including the facts that manual compression is unnecessary and that this elastography is coupled to conventional ultrasound equipment, thus also enabling analysis of other organs.

Elastography and other noninvasive methods have been used over the short term (one to five years) on patients undergoing liver transplantation, in order to determine the degrees of fibrosis.^8^^,^^9^^,^^11^^,^^21^ Determination of the degrees of hepatic fibrosis in liver transplant patients is crucial for early detection of fibrosis and for making decisions on the therapeutic approach to be adopted.^1^

## OBJECTIVE

The current study aimed to evaluate liver elasticity in patients who had undergone liver transplantation at least one year earlier, by means of ARFI elastography, correlating the results with the histological analysis and determining a cutoff value for ARFI results by comparing patients presenting fibrosis grades 0 and 1 with those presenting grades 2 to 4. 

## METHODS

### Study design, location and ethics

In this cross-sectional observational study, we analyzed patients operated on for liver transplantation in the Department of Gastroenterology, Liver Transplantation and Surgery of the School of Medicine of the University of São Paulo. During the period between April 2012 and March 2015, all patients with an indication for liver biopsy were recruited for ARFI evaluation. The research project was approved by the Ethics Committee for Analysis of Research Projects (CAPPESQ), and the patients signed informed consent forms.

### Participants

All the patients who had been consecutively operated for liver transplantation at our institution between 2002 and 2010 and who had a clinical indication for biopsies, and whose transplantation had been performed more than one year before the date of our reassessments, were recruited for participation in this study, in a convenience sample. All of these patients underwent elastography. Patients who presented post-transplantation vascular and biliary complications were excluded.

### Procedures: biopsy, elastography and laboratory tests

The biopsy, elastography and laboratory tests were carried out on the same day, or with not more than 15 days between the procedures. All the ultrasound scans were performed by the same radiologist (JS), and all the anatomopathological exams by the same pathologist (VA). The radiologist and the pathologist were blinded to each other's evaluations and to the patients' laboratory results.

Blood samples were also taken from the patients (not more than 15 days before or after the biopsy and the ARFI examination), to evaluate the following variables: aspartate aminotransferase (AST), alanine aminotransferase (ALT), platelets, albumin, direct and indirect total bilirubin, blood glucose, insulin, Homa index, total cholesterol and fractions and triglycerides. The biochemical reference values were defined in accordance with the ranges of the institution's biochemistry laboratory.

The liver biopsies were collected subcutaneously,^3^^,^^4^ guided by ultrasonography, using needles of 1.2-1.4 mm in diameter. The length of the liver biopsy tissue sample was at least 1.5 mm, with a minimum of 10 port spaces in all cases. The biopsy fragments were immediately fixed in formalin.

**Chart 1:**
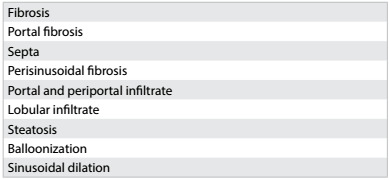
Histological variables analyzed by means of liver biopsy

The inflammatory activity was graded and the fibrosis was staged in accordance with the criteria of the METAVIR Cooperative Study Group^12^ classification and other histological parameters (Chart 1), for each liver biopsy. According to the METAVIR classification, patients staged as F0 or F1 were considered to have no fibrosis or minimal fibrosis. Significant fibrosis was defined as stages F2, F3 and F4. Patients classified as F4 were considered to have hepatic cirrhosis. For this study, the patients were divided in two groups: the F0-F1 group and the F2-F3-F4 group, given that patients with fibrosis grade 2 and above may require treatment, depending on the disease etiology.

The patients fasted for six hours prior to the elastography examination. The equipment used on all the patients was the Siemens S2000 with CH41 transducer (Siemens Healthcare, Ultrasound Business Unit, Mountain View, CA, USA). Twenty hepatic elasticity acquisitions were performed, consisting of 10 in segment V and 10 in segment VIII, with the patient in dorsal decubitus or in left lateral decubitus. The patients held their breath for 5 seconds for each acquisition. The region of interest (ROI) was established in segments 5 and 8, with distances of 3 to 5 cm from the skin in all patients. The results from the measurement acquisitions were expressed in meters per second.

### Statistical analysis

The MS-Excel electronic worksheet (Microsoft Office 2010 version) was used to organize the data, and IBM SPSS (Statistical Package for the Social Sciences), version 22.0, was used for the analysis. A significance level of 5% (P < 0.05) was used.

For inferential statistics, the measurements from ARFI elastography were compared between the fibrosis groups using the Mann-Whitney test. The Kruskal-Wallis and Mann-Whitney tests were used on the ARFI elastography measurements and groups were formed according to the fibrosis scores from the different hepatic regions. The ARFI elastography was analyzed via the median and the mean of the replicates. Cutoff values for elastography were investigated using receiver operating characteristic (ROC) curves.

## RESULTS

During the study period, 33 patients who had undergone a liver transplant more than one year previously, between 2002 and 2010, were included and subjected to liver biopsy and elastography examinations. The original indication for transplantation had been cirrhosis due to the hepatitis C virus in 21 cases (63%) and other causes in 37% (Table 1). Among these 21 patients, 8 were alcohol users. The ARFI elastography was performed successfully in all cases. No complications from the liver biopsy were registered.

**Table 1:**
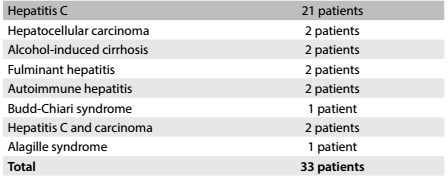
Indications for liver transplantation

Twenty-two of these patients were men. The patients' average age was 55 ± 11 years (mean 49.7; minimum 21; maximum 74). The mean body mass index was 26.2 kg/m^2^ (range: 16.3-32.7).

The anatomopathological results from the liver biopsies showed absence of fibrosis (stage F0) in 10 patients (out of 33, 30.3%), stage 1 fibrosis (F1) in 11 patients (33.3%), stage 2 fibrosis (F2) in eight patients (24.2%), stage 3 fibrosis (F3) in four patients (12.1%) and no cases of stage 4 fibrosis (F4; cirrhosis). The mean ARFI result was 1.11 ± 0.14 for patients staged as F0; 1.21 + 0.28 for those who were F1; 1.36 + 0.35 for patients in the F2 group; and 1.57 + 0.47 for F3 patients. There was no significant difference between groups for ARFI values (P = 0.10). There were significant associations between the fibrosis classification and the number of septa, portal fibrosis, perisinusoidal fibrosis, centrilobular fibrosis and periportal and portal infiltrate (P < 0.05). However, steatosis observed in the histological examination did not show any correlation with fibrosis stage according to ARFI (P > 0.05).

The fibrosis classification groups were then regrouped: FO-F1 (with 21 of the 33 cases, 63%) and F2-F3-F4 (with 12 cases, 37%). The ARFI value was significantly different between these two subgroups: 1.16 + 0.22 (median 1.12, minimum of 0.91 and maximum of 1.99) versus 1.43 + 0.38 (median of 1.38, minimum 0.84 and maximum 2.22), respectively.

The best cutoff value for ARFI that was able to differentiate F0-F1 from F2-F3-F4 was 1.29 m/s, which presented sensitivity of 0.66 (95% confidence interval, CI: 0.50 to 0.83) and specificity of 0.85 (95% CI: 0.72 to 0.97). The area under the ROC curve was 0.74 (95% CI: 0.55 to 0.94). Table 2 shows the accuracy of ARFI at the different stages of fibrosis.

**Table 2:**
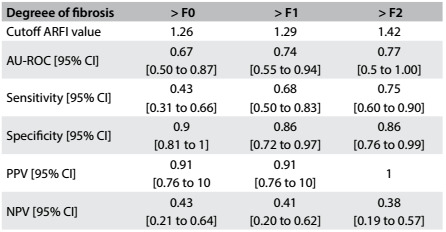
Cutoff values for acoustic radiation force impulse (ARFI) elastography differentiating each group of patients according to the degree of fibrosis, evaluated by means of biopsy, with sensitivity, specificity, positive predictive value (PPV) and negative predictive value (NPV)

**Figure 1:**
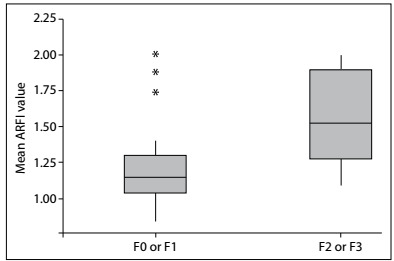
Boxplot of the results from acoustic radiation force impulse (ARFI) elastography regarding the general degree of fibrosis in the two groups of patients, with or without fibrosis.

**Figure 2:**
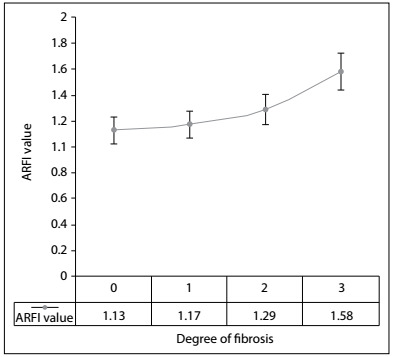
Analysis on the acoustic radiation force impulse (ARFI) values to detect a cutoff point that separates patients with and without fibrosis.

The Kruskal-Wallis test demonstrated statistical differences between the two groups, with P = 0.02 for the general degree of fibrosis (Figure 1), P = 0.031 for septa and P = 0.027 for centrilobular fibrosis. The cutoff point between the groups F0-F1 and F2-F3 was 1.29 m/s (Figure 2).

There was no statistical difference between the values from the ARFI elastography between the patients with hepatitis C and those with other hepatic diseases.

## DISCUSSION

The prospect of a reduction in the number of biopsy procedures performed on operated patients is very important, both for patients and for hospitals, because of the risk of the invasive examination and the cost. Therefore, this study strove to contribute through presenting an alternative to liver biopsy for evaluating post-transplantation liver elasticity.

New noninvasive diagnostic methods have arisen for evaluating the degree of liver fibrosis over recent years: serological tests, elastography and magnetic resonance imaging. Hepatic elastography has been highlighted due to its harmlessness, speed and technical ease of implementation.^13^

Transient elastography was the first widely disseminated method used for evaluating chronic hepatopathy.^17^ Piscaglia et al.^8^ demonstrated that transient elastography produced better results than did biochemical tests for quantifying the degree of fibrosis in patients undergoing liver transplantation because of the hepatitis C virus. In 56 patients, accuracy rates greater than 85% were obtained among those with significant fibrosis.^22^ The study by Piscaglia et al.^8^ contained a description of the importance of future investigations evaluating factors that interfere with hepatic fibrosis, such as hepatic steatosis, perisinusoidal fibrosis and other histological information.

There are few studies on transient elastography performed on transplanted livers, and a meta-analysis only gathered 470 patients.^22^ The studies reviewed showed excellent results regarding identification of cirrhosis and good results in major fibrosis cases. However, findings of low levels of fibrosis from transient elastography may not rule out cirrhosis, and in these cases, biopsy is still needed. The major limitation of transient elastography and other noninvasive methods lies in the interpretation of results from intermediate stages of liver fibrosis. Additionally, transient elastography has the disadvantages of only using A and M modes, and the absence of a B mode precludes evaluation of the liver.

ARFI elastography, the subject of the present study, was the first technique to be coupled with conventional ultrasound equipment, and it has the advantage of viewing both the liver and the measurement site. A meta-analysis on more than 3,000 patients demonstrated that it had high accuracy for quantifying the degree of hepatic fibrosis,^23^ and that its results were similar to those from transient elastography. However, few studies have used ARFI elastography on transplanted livers.^24^

Crespo et al.^25^ examined 87 patients and found that the sensitivity and specificity of ARFI elastography were 76% for F2 and 85% for F4. Wildner et al.^26^ used ARFI elastography on 58 patients who underwent transplantation and showed that the velocities were significantly higher in the patients with advanced fibrosis. In the present prospective study, despite the limited number of patients, two primary results can be noted: firstly, ARFI elastography was able to distinguish between livers transplanted in the group with F0-F1 and those in the group with F2-F3, with a statistically significant difference. There was significant correlation between the velocities obtained and the presence of septa and centrilobular fibrosis.

The current case study was on patients who received transplants due to hepatitis C virus or other forms of chronic hepatopathy. In the analysis on the two fibrosis groups, the underlying disease was not taken into consideration. This was in fact one limitation of this study: its analysis of patients with hepatitis C and other liver diseases together. Another limitation was the low number of patients, which serves as a stimulus for further investigations on this subject. The velocities obtained in the various degrees of fibrosis following transplantation were similar to those described in populations of chronic hepatopathy cases that did not undergo transplantation.^10^^,^^13^

These preliminary results indicated that the values obtained through ARFI elastography were not affected by conditions that could change these values, such as inflammatory reactions or anatomical alterations. Absence or a low number of patients with F4 was expected, as also found by other authors, due to the good results from antiviral therapies. The differences between biopsy and ARFI results may be due to histological analyses performed in different regions of the liver, heterogeneous liver tissue samples and obese patients. We believe that the following factors could possibly lead to wrong diagnostic conclusions: biopsy samples smaller than 1.5 cm with less than 10 portal spaces; heterogeneous liver tissue, with variable fibrosis density; or obese patients, in which the elastography results are false negatives or false positives.

The second conclusion is that a cutoff value of 1.29 meters per second that separates patients with or without significant fibrosis was identified. This may influence antiviral therapy over the short term, because higher velocities indicated by ARFI mean higher degrees of fibrosis, which require antiviral treatment. The follow-up on fibrosis progression could include this noninvasive method in future post-transplantation protocols.

The goal of ARFI elastography is not to replace liver biopsy in all transplanted livers. Other alterations, such as rejections and vascular or biliary abnormalities, usually require a liver biopsy for diagnosis and follow-up.

This study has demonstrated promising results with regard to differentiation of patients with fibrosis grades 0 and 1 from those with grades 2 to 4, through ARFI elastography. Further studies with larger samples of patients are necessary in order to confirm these results and possibly include ARFI in the protocol for evaluating transplantation patients.
